# Pathogen cross-transmission via building sanitary plumbing systems in a full scale pilot test-rig

**DOI:** 10.1371/journal.pone.0171556

**Published:** 2017-02-10

**Authors:** Michael Gormley, Thomas J. Aspray, David A. Kelly, Cristina Rodriguez-Gil

**Affiliations:** School of Energy, Geoscience, Infrastructure and Society, Heriot-Watt University, Riccarton Campus, Edinburgh, United Kingdom; University of British Columbia, CANADA

## Abstract

The WHO Consensus Document on the epidemiology of the SARS epidemic in 2003, included a report on a concentrated outbreak in one Hong Kong housing block which was considered a ‘super-spreading event’. The WHO report conjectured that the sanitary plumbing system was one transmission route for the virus. Empty U-traps allowed the aerosolised virus to enter households from the sewerage system. No biological evidence was presented. This research reports evidence that pathogens can be aerosolised and transported on airstreams within sanitary plumbing systems and enter buildings via empty U-traps. A sanitary plumbing system was built, representing two floors of a building, with simulated toilet flushes on the lower floor and a sterile chamber with extractor fan on the floor above. Cultures of a model organism, *Pseudomonas putida* at 10^6^–10^9^ cfu ml^-1^ in 0·85% NaCl were flushed into the system in volumes of 6 to 20 litres to represent single or multiple toilet flushes. Air and surface samples were cultured on agar plates and assessed qualitatively and semi-quantitatively. Flushing from a toilet into a sanitary plumbing system generated enough turbulence to aerosolise pathogens. Typical sanitary plumbing system airflows (between 20–30 ls^-1^) were sufficient to carry aerosolised pathogens between different floors of a building. Empty U-traps allowed aerosolised pathogens to enter the chamber, encouraging cross-transmission. All parts of the system were found to be contaminated post-flush. Empty U-traps have been observed in many buildings and a risk assessment indicates the potential for high risk cross-transmission under defect conditions in buildings with high pathogen loading such as hospitals. Under defective conditions (which are not uncommon) aerosolised pathogens can be carried on the airflows within sanitary plumbing systems. Our findings show that greater consideration should be given to this mode of pathogen transmission.

## Introduction

The spread of disease via building environmental systems is, on the whole, little understood. Sporadic investigations on pathogen transmission have mostly focussed on *Legionella pneumophila* in air conditioning and water supply systems [1–5] with some additional work being carried out on biofilm formation in sinks and pathogen fallout from flushing toilets [6–9]. While the study of *L*. *pneumophila* transmission from water supply systems is now well understood, principally due to the steady, predictable nature of the water flows involved, this is not the case for the transmission of pathogens from the sanitary plumbing system. These systems are characterised by unsteady and turbulent wastewater flows due to random discharges from sanitary fittings such as sinks, baths, showers and toilets. These flows, in turn, induce unsteady transient airflows inside the plumbing pipe network. Wastewater flows inside the sanitary plumbing system lead to pressure fluctuations which can compromise the fragile water trap seals (U-traps) which form the only protection between the sanitary plumbing system and the people within buildings.

**Fig 1:**
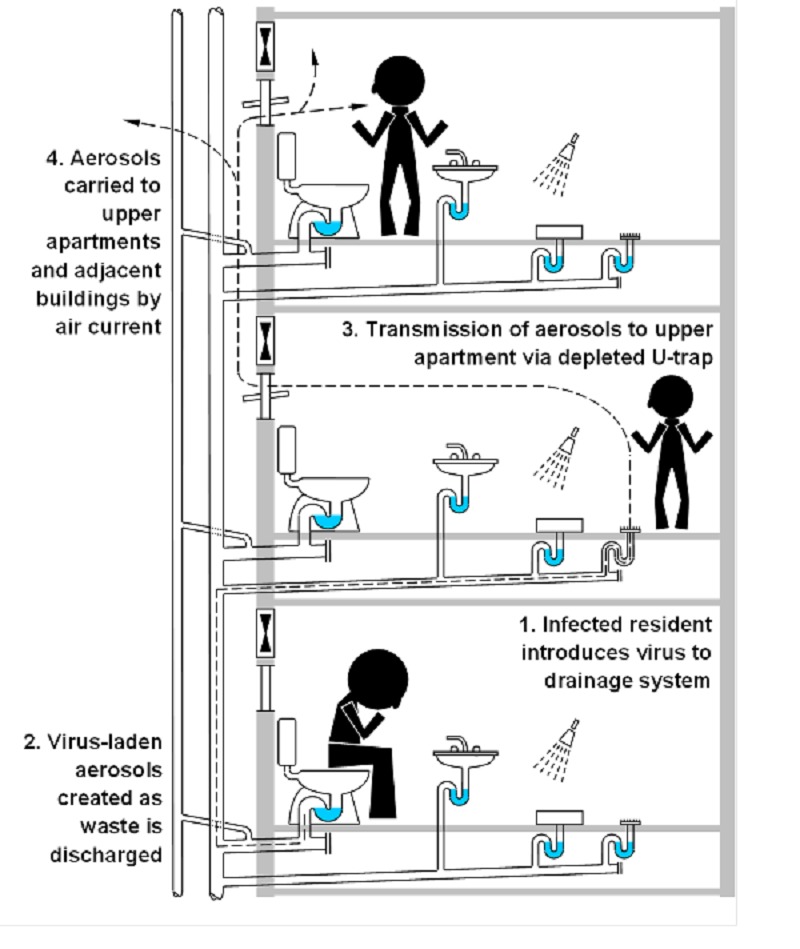
SARS transmission route at Amoy Gardens via the sanitary plumbing system.

Since the SARS outbreak in 2002/2003 [10], there has been growing concern regarding the role that the sanitary plumbing system played in the transmission of the virus. The World Health Organisation (WHO) Consensus Document [11] on the epidemiology of the outbreak included a report on the transmission of SARS in one particular housing block in Hong Kong (Amoy Gardens) which was considered as a ‘super-spreading event’. A total of 341 cases of SARS were reported at Amoy Gardens, resulting in 42 deaths. One of the reasons cited for this high infection rate was the spread of the virus via the building’s sanitary plumbing system. The WHO conjecture stated that *“dry U-traps in bathroom floor drains provided a conduit for contaminated sewage droplets to enter households*. *A significant virus load had built up in the sewer system as an increasing number of SARS cases with diarrhoea excreted virus*. *Virus was aerosolized within the confines of very small bathrooms and may have been inhaled*, *ingested or transmitted indirectly by contact with fomites as the aerosol settled”* [11]. The U-traps (particularly in floor gullies) were found to be depleted of water, thus having lost their sealing function and providing an open connection between the sanitary plumbing system and different apartments within the building. As the number of SARS cases increased, the sanitary plumbing system became a reservoir for the virus due to diarrhoeal excretion. The WHO hypothesis followed that the virus became aerosolised when discharged into the sanitary plumbing system which provided a conduit for virus-laden aerosols to enter apartments via depleted U-traps. This process was exacerbated by the naturally occurring airflows within the sanitary plumbing system and the negative pressures within bathrooms as a result of extract fans. The combination of these factors presented a pathway for pathogen transmission heretofore unexplored. The process is illustrated in Fig 1.

**Fig 2:**
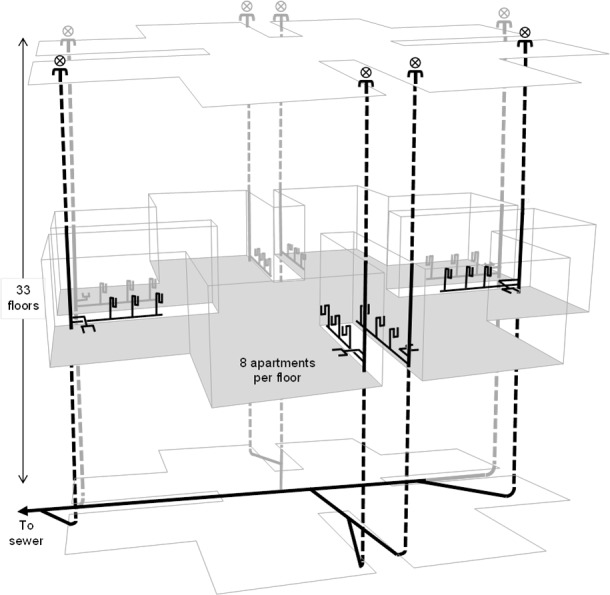
Interconnectedness of whole building.

The interconnectedness of the sanitary plumbing system, and the airflow communication pathways that exist between all system points are rarely understood and almost never considered within the context of pathogen transmission. Fig 2 illustrates the interconnected nature of the sanitary plumbing system for a building similar to Amoy Gardens. Aerosols and droplets carrying infectious agents can be carried in airflows [12,13] and so an understanding of the airflows within both the sanitary plumbing system and within the building itself is imperative in order to track transmission routes.

While research into the potential mechanisms of the SARS transmission at Amoy Gardens proved that there was an airflow path between the sanitary plumbing system and different parts of the building when the U-trap had no water seal [14,15], it was only hypothesised that pathogens could be transmitted within these airflows and could deliver an infective exposure dose. This paper sets out to test the WHO hypothesis by qualitatively and quantitatively tracking the transmission of a model organism chosen to represent pathogens generally within the airflows of a full scale pilot test-rig representing a two storey sanitary plumbing system designed to current European standards [16]. The data gained from the pilot test-rig is supplemented with on-site observational data of sanitary plumbing defects collected over many years by the Authors to identify the risk factors of transmission.

## Methods

### Full scale pilot test-rig setup

**Fig 3:**
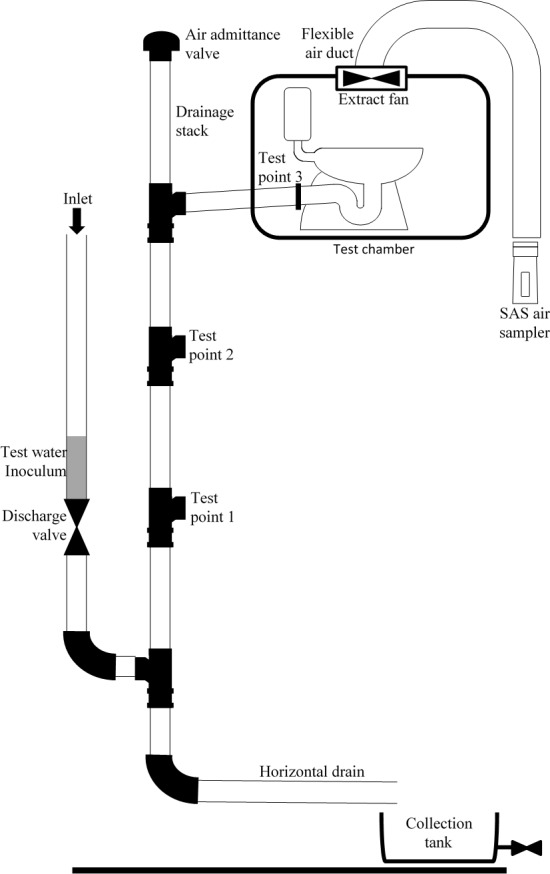
Full scale 2-storey pilot test-rig.

A full scale pilot test-rig was constructed to conform with BS EN 12056 [16]; the British and European Standard for the design and construction of sanitary plumbing systems. The two floor test rig is shown in Fig 3 and was constructed with standard 100 mm diameter PVC-u rigid pipe and consisted of a main vertical pipe (stack) connected to a horizontal drain. The system included a receptacle pipe to hold an inoculum which was discharged into the main vertical stack at the lower floor via a toilet flush simulator valve. The valve was operated to simulate a flush similar in characteristics to a standard drop valve toilet (a typical toilet flush volume in the U.K. is 6 litres, however other legacy toilets have flush volumes of 7.5, 9 and 13 litres, these are also typical volumes for toilets globally). The simulator had the capacity for either a single standard 6 litre flush or multiple flushes [17]. The flushed inoculum was then directed to a collection tank. At the upper floor, a test chamber was constructed to represent a bathroom containing a toilet, of which the U-trap could be emptied of water, and an extract fan connected to a flexible duct at the end of which air samples could be taken. A typical U-trap for a toilet will contain 1–1.5 litres of water and for sinks, baths, showers and other fixtures the volume of water will be up to 400ml depending on the type and model. Two further test points (Test Point 1 and Test Point 2) were located between the lower and upper floors, and a further test point (Test Point 3) was located within the horizontal pipe within the test chamber to allow air samples to be taken from within the system.

Airflow rates within the test-rig were adjusted using the extract fan to correspond to those found in real systems.

While there is currently very little literature on the airflows found within sanitary plumbing systems [18], one key piece of literature investigated the airflow within the sanitary plumbing system at Amoy Gardens [11] and found the airflow rate at a bathroom floor drain to be 28 ls^-1^ with a direction of flow from the sanitary plumbing system into the bathroom. Investigations by the Authors [19,20] into the airflow characteristics within sanitary plumbing systems of a three-storey house found similar results with peak airflow rates ranging from 18·6 ls^-1^ to 26·7 ls^-1^. Smoke tests of this three-storey system showed that plumes of air would enter the bathroom when the U-trap was missing. Airflow rates within the test-rig were varied up to a maximum of 29.8 ls^-1^ or operated without airflow.

There are two main ways in which airflow can occur within a sanitary plumbing system:

1. As a result of wastewater flowing down the vertical stack. This has the effect of entraining an airflow into the system from the top. The direction of this airflow is from top to bottom.
2. Air flowing out of the main sewer and through the vertical stack to the exit at the top of the building. This airflow depends on many factors including; sewer activity levels, temperature differentials between top and bottom of the vertical stack, chimney effects and wind shear across the top to the vertical stack. The direction of this airflow is from bottom to top.

The airflow of most interest in this study is defined in ii) above. This is because the airflows from i) will flow from the building into the sewer. The flows described in ii) can travel from the sewer and into the building, and so have potential to carry aerosolised pathogens with it. The upward flow of air will travel the shortest route towards atmospheric pressure and an empty U-trap provides such a route.

Only two studies have published relevant data on airflows from sanitary plumbing systems:

1. Amoy Gardens (a50 storey building) was found to be 28 ls^-1^
2. The 3storey house was recorded 18.6 and 26.7 ls^-1^

The task of inducing a flowrate in the pilot test-rig was difficult to do precisely (small variations in system operation and the random nature of some of the variables meant that exact flow rates could not be repeated. It was decided to set 3 ranges of airflow rate in order to carry out test repeats. The airflow rates were classified as either low (< 20 ls^-1^), medium (20 ls^-1^–27 ls^-1^) or high (>27ls^-1^).

#### *Pseudomonas putida* culture preparation

Due to the preliminary nature of this work, a class 1 non-genetically modified bacterium, *Pseudomonas putida* KT2440 [21], was used as a reliable model organism in 10 experiments to assess pathogen cross contamination. Pure colonies were picked from agar plates, inoculated into Erlenmeyer flasks containing tryptone soya broth and incubated overnight at 30°C with orbital shaking. The resulting stock culture was diluted in 0·85% (w/v) NaCl to provide the inoculum. An aliquot of this culture was used to determine cell concentration. For experiments 1–5, an inoculum of 10^7^−10^9^ cfu ml^-1^ was used. For experiments 6–10, a lower inoculum concentration (10^6^ cfu ml^-1^) was used to reduce number of cells reaching the active air sampler and oversaturating.

#### Monitoring approaches

A) Passive sampler (Experiments 1, 2, 4–7)

**Fig 4:**
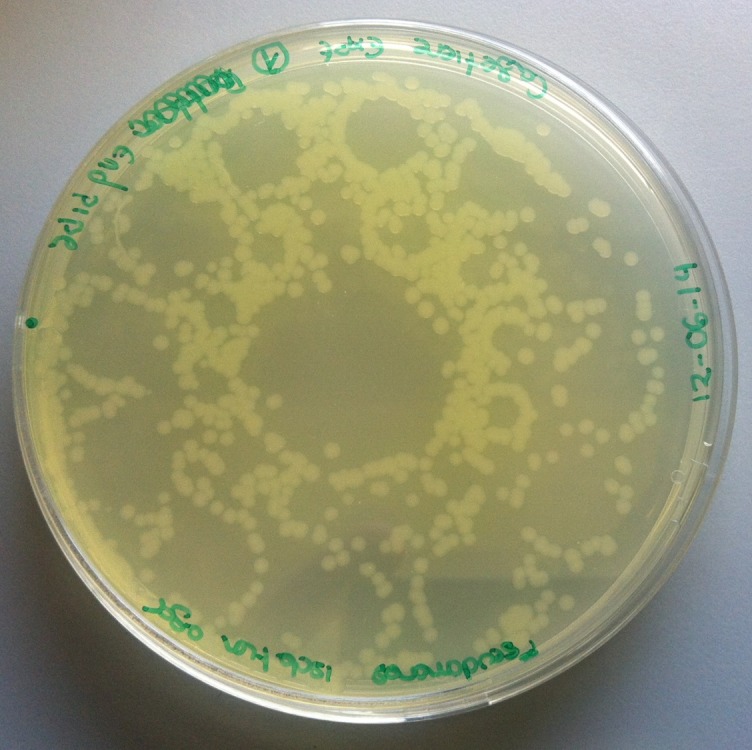
Pattern and number of viable *Pseudomonas putida* KT2440 at the end of the horizontal pipe (experiment 9).

Stainless steel ‘upper discs’ from domestic cafetieres (or coffee presses) were adapted as novel passive air samplers (i.e. not described in BS EN ISO 14698–1:2003) [22] and inserted into test points indicated in Fig 3. Each test point was carefully sealed whether in use or not. Each sampler was sterilised in sealed bags by autoclaving prior to use. After use, each sampler was carefully removed from its test point and pressed onto a standard 90 mm diameter Pseudomonas Isolation Agar (PIA; Sigma Aldrich) plate (resulting growth pattern as illustrated in Fig 4). The plates were incubated overnight at 30°C.

B) Swabs and contact plates (Experiments 1–2, 6- and 7)

Swabs were used to take surface samples during Experiment 1, after which, 55 mm diameter (23·8 cm^2^ area) contact plates were used. For Experiment 2, non-selective nutrient plates were used, after which, PIA plates were used for subsequent Experiments (as required). Contact plates were touched once onto test chamber surfaces and incubated for 24 h at 30°C.

C) Active air sampler (Experiments 2–5, 8–10)

A Surface Air System (SAS Super IAQ; International PBI S.p.A) indoor air quality monitor, containing a single 90 mm diameter PIA plate, was setup at the end of the flexible duct connected to the extract fan. The monitor was set to sample 250 litres of air at a rate of 100 litres per min prior to each experiment. The PIA plate was replaced and air sampling repeated at the same time as the test inoculum (containing *P*. *putida* KT2440) was flushed into the system. PIA plates were incubated for 24 h at 30°C before colony counting.

#### Toilet U-trap tests (Experiments 8, 9 and 10)

A standard domestic toilet was sterilised by autoclaving and fitted into the test chamber (see Fig 3). A partial water trap was prepared using 180 ml (Experiment 8) and 350 ml (Experiment 10) sterile 0·85% (w/v) NaCl, respectively. An empty U-trap was used for Experiment 9. After Experiments 8 and 10, the partial water trap solution was collected and vacuum filtered through a sterile 0·45 μm filter. The filter was incubated on a PIA standard (90 mm diameter) plate. Colonies were counted after 24–72 h.

**Fig 5:**
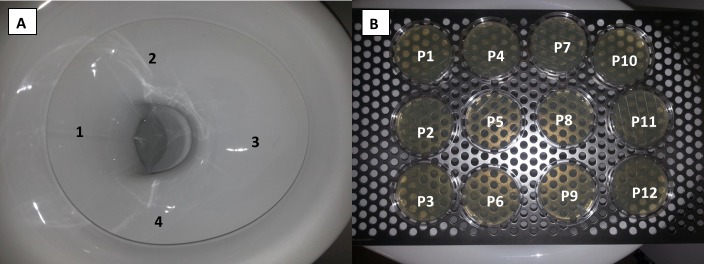
**Toilet bowl (A) and toilet seat (B) surface sampling locations.** Front of toilet to right of both images.

For experiments 9 and 10, a perforated steel plate was sterilised in sealed bags by autoclaving prior to use and positioned flat on the toilet bowl (Fig 5B). After each experiment, the plate was reversed and sampled in a grid like fashion using PIA contact plates (Fig 5B).

#### Test- rig decontamination

The test-rig was fully decontaminated between each experiment using a biochlor® solution flush followed by rinsing with mains tap water. The rig sterility was checked using the active air sampler and PIA plates and found to be effective. The chamber was decontaminated by surface sterilisation and confirmed clean with the use of PIA contact plates.

#### On-site observations of defective U-traps

In conjunction with the full scale 2-storey pilot test-rig experiments, observation data from previously completed on-site research were collated in terms of observed depleted U-trap occurrence. The data was collated by building type and location, size of building and location of depleted U-trap within the building. The extent of the depletion and a likely cause was also assessed.

## Results and discussion

### Microbiological results

The microbiological results from the experiments using the full scale pilot test-rig are shown in Tables 1 and 2. Experiments 1 to 7 are those with no U-trap connected at the upper floor and an empty test chamber, whereas, Experiments 8–10 include a toilet connected at the upper floor in the chamber as illustrated in Fig 3.

**Table 1:** Empty chamber experiments. Reported values are colony forming units (CFU) with CFU/cm^2^ in parenthesis.

Expt.	Airflow	Inoc.	Inoc.	Passive sampler CFU			Chamber surface										Active air
no.	rate	vol (l)	CFU				CFU (CFU/cm^2^)										sampler
			(ml^-1^)	TP1	TP2	TP3	Pre-flush					Post-flush					CFU
							B	L	T	R	F	B	L	T	R	F	
1	HIGH	6+8+6	1·93x10^8^	752	51		0	0	0	0	0	0	0	0	0	0	
2	HIGH	8+3	9·13x10^9^			45	0	0	0	0	2 (0·08)	0	0	1 (0·04)	1 (0·04)	2 (0·08)	64
3	HIGH	6	1·20x10^7^														197
4	HIGH	6	3·28x10^6^		736	476											248
5	LOW	6	9·10x10^6^		748	484											282
6	n/a	6	7·33x10^6^	Pos^#^		0	0	0	0	0	0	2 (0·08)	0	0	0	0	
7	n/a	6	4·48x10^6^		2	1	0	0	0	0	0	2 (0·08)	0	0	0	0	

*Air flow rates (ls^-1^) equate to; low (<20), medium (20–27) and high (>27)

B = bottom wall, L = left wall, T = top wall, R = right wall, F = front wall, TP = Test Point

Blank cells indicate where testing was not carried out or sample locations were unavailable

^#^Passive sampler splashed by flush–count not possible.

**Table 2:** Chamber with toilet experiments. Reported values are colony forming units (CFU) with CFU/cm^2^ in parenthesis.

Expt.	Airflow	Inoc.	Inoc. CFU	U-trap	Chamber Surface CFU (CFU/cm^2^)					Toilet Surface CFU (CFU/cm^2^)				Toilet seat height												Active air
no.	rate*	vol. (l)	(ml^-1^)	water (ml)	Post-flush					Post-flush				Post-flush												sampler
					B	L	T	R	F	1	2	3	4	P1	P2	P3	P4	P5	P6	P7	P8	P9	P10	P11	P12	CFU
**8**	Medium	6	3·57x10^8^	180	0	0	2 (0·08)	1 (0·04)	2 (0·08)	6 (0·25)	6 (0·25)	18 (0·76)	4 (0·17)													510
**9**	Medium	6	8·39x10^8^	0										-	-	-	-	-	-	-	-	-	+	+	-	457
**10**	Low	6	7·90x10^6^	350										+	-	-	+	+	+	+	+	+	+	+	+	318

*Air flow rates (ls^-1^) equate to; low (<20, medium (20–27) and high (>27)

B = bottom wall, L = left wall, T = top wall, R = right wall, F = front wall

Blank cells indicate where testing was not carried out or sample locations were unavailable.

Collectively, the 10 experiments confirm that cross-transmission of viable bacteria can occur between adjacent floors of a sanitary plumbing system. The flushing of a toilet containing wastewater on a lower floor contaminated the room (test chamber) on the upper floor. This occurred both with an induced upward airflow (Experiments 1 to 5, and 8–10) and without (Experiments 6 and 7), however, the cross-contamination was less severe in the absence of an airflow based on passive air sampler data. Finally, based on passive air sampler data, Experiments 4 and 5 which had comparable inoculum concentrations show similar numbers of bacteria at test points 2 and 3. This result is supported by active air sampler data at the extractor ducting exit.

Two different approaches were assessed for detecting and quantifying the model bacterium, *P*. *putida* KT2440, in the test chamber (Tables 1 and 2). In Experiment 1, swabs were used to assay the test chamber surfaces, however, no CFUs formed on plates. The swabs were substituted for non-selective media contact plates in Experiment 2. Although CFUs were reported after this experiment on three walls of the test chamber, two CFUs (of differing colour/morphology to *P*. *putida* KT2440) were also found on one wall before the experiment (after alcohol surface cleaning). However, these subsequently did not grow when streaked onto PIA and were assumed environmental contaminants. PIA contact plates were used in subsequent experiments to eliminate the need for a sub-culturing confirmatory step(s). The contact plates showed contrasting results depending on whether or not airflow was applied. Specifically, in Experiments 2 and 8, which were carried out with an applied upward airflow, CFUs appeared on plates which sampled from the top, right and front walls of the test chamber. By contrast, in Experiments 6 and 7, carried out with no induced airflow, CFUs were only found on the bottom surface sample. This confirms that even in the absence of an applied airflow, cross-transmission into the test chamber was achievable.

Three experiments (8, 9, and 10) were carried out incorporating a pre-sterilised toilet within the test chamber connected at the upper floor. Experiment 8 showed that with a partially filled U-trap, cross-contamination was apparent on the toilet itself, on the test chamber surfaces, and at the end of the flexible duct connected to the extract fan. On a surface area basis, the contact plates suggest the toilet contamination was higher than the test chamber and focused towards the front of the toilet bowl (position 3 in Fig 5A, see Table 2) and, therefore, was probably influenced by the draw of the extract fan. In Experiments 9 and 10, a second novel passive sampler method was trialled involving a perforated plate placed at toilet seat height. After these experiments the perforated plate was over-turned and sampled using contact plates. However, the plate was wet after each experiment and likely to have affected the numbers of bacteria recovered from this surface; results are reported in Table 2 as presence/absence rather than actual numbers. As with the toilet surface sampler from Experiment 8, the perforated plate sampling suggested a focus towards the front of the toilet bowl (P10 and P11 in Fig 5B and Table 2).

As well as passive air and surface sampling, an active air sampler was used to quantify bacteria transmitted through the entire system by positioning it at the exhaust termination of the flexible duct connected to the extract fan, see Fig 3. CFUs formed on PIA plates from all experiments using the active air sampler, confirming the cross-transmission of bacteria through the entire sanitary plumbing test-rig: from the flushing of contaminated wastewater at the lower floor and into the test chamber, as evidenced by the passive and surface sampling methods, and then into the extract ventilation system. Although CFU counts are given for the active air sampler in Tables 1 and 2, these are considered as semi-quantitative for several reasons. Firstly, active air sampling was used in conjunction with passive sampling in several experiments (Experiments 2, 3, 4, 5, 9, and 10) which will have impacted on the number of cells flowing through the system. Secondly, the active air sampler had a fixed flow rate of 100 litres per minute (i.e. 1·67 ls^-1^) and was, therefore, considerably lower than the peak airflow rate within the test-rig (29·8 ls^-1^) meaning that cells will have been lost by bypassing the active air sampler. In addition to this, the different airflow velocity in the test-rig may have affected the sampling flow rate of the active air sampler and, therefore, impact velocity of the active air sampler itself. Assuming this was the case, a higher impact velocity (more than double the sampler’s optimal at the highest flow rate) could easily have resulted in lethal injury to bacterial cells and subsequent under reporting [23]. Finally, in Experiments 8 and 9, a high surface density of CFUs on the agar plate may have resulted in masking of colonies and subsequent under reporting [24].

### On-site observations of defective trap seals

Observation data from previously completed on-site research carried out over many years were collated and suggest that U-trap depletion is not uncommon. Table 3 below shows examples of buildings where the Authors have observed depleted U-traps or have been involved in efforts to alleviate recurring problems in specific buildings (these efforts include re-design of ventilation and pressure alleviation systems, and replacement of water traps with waterless, sheath versions). Depleted U-traps have been found in buildings in many countries and in many different types of building. It is important to note that the majority of these buildings are high occupancy and that two of the buildings observed to suffer from depleted U-traps in the UK are hospitals.

**Table 3:** Observations of depleted U-traps within different locations and building types.

Location	Building type	No. of floors	U-trap location	Partially depleted	Fully depleted	Likely cause
UK	Hospital	5	Bathroom	√		Evaporation/ under use
UK	Hospital	5	Plant room		√	Evaporation/ under use
UK	University campus	5	Plant room		√	Evaporation/ under use
UK	Office building	8	Basement		√	Evaporation/ under use
Ireland	Residential	1	Bathroom		√	Evaporation/ under use
USA	Hotel	10	Bathroom		√	Pressure transients
China	Office building	7	Public toilets	ID		Pressure transients
Hong Kong	Residential	50	Bathroom		√	Evaporation/ under use
UK	Hospital	7	Wards		√	Unknown

ID = Intermittent depletion.

## Discussion

The experiments prove that pathogens can be transmitted from one part of a building to another on sanitary plumbing system airstreams.

The U-trap is designed to provide a physical barrier between the sanitary plumbing system and building in order to prevent cross-transmission of airstreams. However, it is vulnerable to particular environmental and system conditions that make it susceptible to depletion. Table 4 details the hazards that influence the risk of U-trap depletion.

**Table 4:** Hazards of water trap seal depletion.

Hazard	Risk to U-trap	Cause
Under-use	Evaporation	Vacant/under-utilised building
High ambient temperature	Increased rate of evaporation	Local climate
		Internal heat gains
		Poor design or construction
		Over-loaded system
Excessive system	Self- siphonage or	Tall building *(> 30 storeys)*
pressure transients	induced-siphonage	External air pressures
		(wind shear/sewer surcharge)
		Chimney effect

If an appliance is under-used then the water seal within the U-trap can simply evaporate over time. The rate of evaporation under normal ambient conditions in the UK has been found to be in the region of 3 mm per week [25]. A 50 mm water seal would, therefore, fail after a period of 17 weeks of non-use. Increased rates of evaporation occur due to higher ambient temperatures. Anecdotal evidence gathered by the authors has found that the water within a 50 mm floor gulley trap located within a boiler room evaporated completely within a 24 hour period.

The water seal is also vulnerable to fluctuations in air pressure that can propagate throughout the system as pressure transients. Excessive negative pressures generated by discharging appliances or adjacent appliances, can lead to trap depletion by self-siphonage or induced-siphonage, respectively. The propagation of excessive positive pressures within the system can be large enough to completely displace the water seal into the appliance [26,27,28], leaving the trap either wholly or partially depleted. In some cases, even though the trap is not noticeably depleted, positive pressures can push air from the sanitary plumbing system through the water seal as air bubbles and into the building. Excessive system pressure transients can be caused due to poor design and/or construction, or when a system is simply over-loaded. They are also prevalent in tall buildings as design guides tend to have limited understanding of sanitary plumbing system operation in buildings over 30 storeys. External factors, such as wind shear over the stack termination and sewer surcharge, can also generate excessive pressure transients capable of depleting the water seal.

Since a U-trap is effectively a manometer, the important parameter relating to its retention is the water seal depth (not volume of water). A typical water seal depth for a sink, bath, shower is 50 mm and for a toilet it is 75 mm (in some hospital isolation wards these can be as large as 200 mm) Therefore a negative pressure in excess of 50 mm water gauge (wg) will suck water out of a sink U-trap causing a direct breach of the seal between the sanitary foul system side and the room and a positive pressure wave in excess of 50 mm will blow through into the room via a sink. In some cases partial depletion or partial flow through (bubble through) can occur which allows foul air to enter the room while the U-trap may look intact afterwards. Limiting pressure fluctuations forms the basis of much of the design of a sanitary plumbing network and while great efforts are made to reduce the likelihood of this, transient generation and propagation is inevitable (particularly in larger systems where overload is possible). Air pressure surges in excess of 50 mm (both negative and positive) are not uncommon and while efforts are made to limit these by introduce air admittance valves and P.A.P.A’s into the system, localised pressure surges and hence depleted U-traps, are inevitable on large systems[5, 26,27,28].

**Fig 6:**
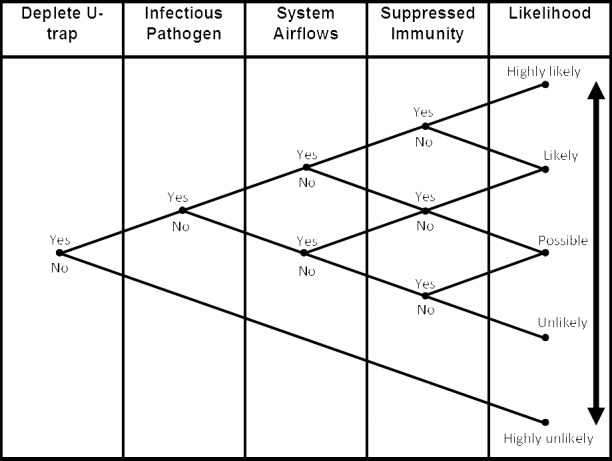
Transmission of infection from sanitary plumbing systems: risk factors and likelihood (adapted from OECD, 2005 [29]).

The likelihood of a building occupant becoming infected through transmission from the sanitary plumbing system is dependent on four main variables: (i) the presence of a depleted U-trap; (ii) the presence of infectious pathogens within the sanitary plumbing system; (iii) adequate system airflows to transmit the infectious pathogens; and (iv) the susceptibility of the occupant to those infectious pathogens, heightened due to immunity suppression. The likelihood of infection is increased by the affirmative presence of each risk factor, see Fig 6. Taking Amoy Gardens as an example, the presence of depleted U-traps, the presence of the SARS virus within the sanitary plumbing system, sufficient airflows to transmit the virus to adjacent apartments, but no residents with a suppressed immunity shows, in that situation, the transmission of SARS was likely.

## Conclusions

The conclusions from our work are;

- Under defect conditions a transmission route exists within the sanitary plumbing system which could allow pathogens to move from one part of a building to another.
- Our experiments in a full-scale pilot test-rig of a 2 storey sanitary plumbing system showed that the turbulence caused by a toilet discharge was sufficient to aerosolise a model organism (*P*. *putida*) which was then carried on airstreams and contaminated pipework, toilet bowl, room surfaces and extract fan system.
- The passive sampling techniques developed in this research were effective as a semi-quantitative approach for the sampling of a model organism in an airflow.
- The air flow rates used in these experiments were typical of those found in real buildings. Our findings indicate that whilst the extent of organism transport was negligible when there was no induced upward airflow, cross-transmission between floors did occur for all experiments where there was an induced airflow (>16.73 ls^-1^).
- We have developed a qualitative risk assessment tool for building managers and maintenance personnel to use in order to assess the risk of a person becoming ill as a result of this mode of cross transmission.
